# Exploring the Disease-Associated Microglia State in Amyotrophic Lateral Sclerosis

**DOI:** 10.3390/biomedicines11112994

**Published:** 2023-11-08

**Authors:** Carlota Jauregui, Idoia Blanco-Luquin, Mónica Macías, Miren Roldan, Cristina Caballero, Inma Pagola, Maite Mendioroz, Ivonne Jericó

**Affiliations:** 1 Neurology Department, Hospital Universitario de Navarra (HUN), IdiSNA (Navarra Institute of Health Research), 31008 Pamplona, Spain; carlotajaureguilarranaga@gmail.com (C.J.); inmaculada.pagola.lorz@navarra.es (I.P.); 2 Neuroepigenetics Laboratory, Navarrabiomed, Universidad Pública de Navarra (UPNA), IdiSNA (Navarra Institute of Health Research), 31008 Pamplona, Spain; idoia.blanco.luquin@navarra.es (I.B.-L.); monica.macias.conde@navarra.es (M.M.); miren.roldan.arrastia@navarra.es (M.R.); 3 Department of Pathology, Hospital Universitario de Navarra (HUN), IdiSNA (Navarra Institute of Health Research), 31008 Pamplona, Spain; mc.caballero.martinez@navarra.es; 4 Neuromuscular and Neuron Motor Diseases Research Group, Navarrabiomed, IdiSNA (Navarra Institute of Health Research), 31008 Pamplona, Spain

**Keywords:** neurodegeneration, amyotrophic lateral sclerosis, neuroinflammation, disease-associated microglia, MS4A, CD33

## Abstract

Background: Neuroinflammation, and specifically microglia, plays an important but not-yet well-understood role in the pathophysiology of amyotrophic lateral sclerosis (ALS), constituting a potential therapeutic target for the disease. Recent studies have described the involvement of different microglial transcriptional patterns throughout neurodegenerative processes, identifying a new state of microglia: disease-associated microglia (DAM). The aim of this study is to investigate expression patterns of microglial-related genes in ALS spinal cord. Methods: We analyzed mRNA expression levels via RT-qPCR of several microglia-related genes in their homeostatic and DAM state in postmortem tissue (anterior horn of the spinal cord) from 20 subjects with ALS-TDP43 and 19 controls donors from the Navarrabiomed Biobank. Results: The expression levels of *TREM2*, *MS4A*, *CD33*, *APOE* and *TYROBP* were found to be elevated in the spinal cord from ALS subjects versus controls (*p*-value < 0.05). However, no statistically significant gene expression differences were observed for *TMEM119*, *SPP1* and *LPL*. Conclusions: This study suggests that a DAM-mediated inflammatory response is present in ALS, and *TREM2* plays a significant role in immune function of microglia. It also supports the role of *C33* and *MS4A* in the physiopathology of ALS.

## 1. Introduction

Amyotrophic lateral sclerosis (ALS) is a fatal neurodegenerative disease characterized by the progressive loss of upper and lower motor neurons. Neuroinflammation is one of the pathogenic processes presumably involved in the disease, but despite being a consistent feature in ALS, its role has yet to be defined. It is important to deepen our understanding of this feature as it is an interesting therapeutic target for the disease. In addition, a complex signaling network between central nervous system (CNS) resident immune cells (microglia) and peripheral immune cells, including monocytes and T cells, has been reported in ALS and other neurodegenerative disorders [1].

Microglia are the resident immune cells of the brain and contribute to synapse formation, maintenance and immune surveillance [2]. Since its early characterization, different microglial states or patterns have been described, and there is evidence that microglia play an important role in CNS diseases through their ability to orchestrate the inflammatory response by secreting modulatory cytokines or eliminating apoptotic neurons through phagocytic and restorative functions. Apparently, microglia not only respond to disease, but also modulate its course [3].

The hypothesis of a dynamic and dichotomic polarization of microglia between a pro-inflammatory and an anti-inflammatory phenotype [4] is today rather simple and possibly does not adequately reflect the complexity of the physiological role of microglia in CNS diseases, including ALS. The characterization of microglia is currently based on the expression of specific genes that confer a transcriptional identity to these cells [5,6], which can be modified according to local or environmental signals defining different functional states of microglia [7]. Thus, microglia are a heterogeneous cell population with regional differences even under basal conditions, with the change in functional state of microglia being a dynamic process.

Disease-associated microglia (DAM) are one of the different states of microglia described in recent years. DAM has a unique transcriptional and functional signature, originally described in an animal model of Alzheimer’s disease (AD) [8]. Initially, a two-step sequential differentiation model was proposed to explain the transition from homeostatic microglia to DAM [9], in which *TREM2* signaling is required for the induction of the DAM state [10]. However, other single-cell studies in human AD brains revealed a microglial transcriptional signature that partially recapitulated that of the animal model [11,12], but with notable differences [13,14,15]. It is currently controversial to consider that there is a universal signature of the microglial response to neurodegenerative damage in the human brain [16]. Other microglial states have also been described across several disease models, such as the microglial neurodegenerative phenotype (MGnD) [17] and the specific microglial signature in Parkinson’s disease (PD) [18]. To our knowledge, no microglial state has yet been described specifically in ALS.

The aim of this study is to analyze the expression of homeostatic and DAM-related genes in postmortem spinal cord tissue from ALS patients versus control donors because the knowledge of this microglial state is very limited in ALS. We have developed this study as a preliminary work to encourage further studies that will continue to explore the transcriptional identity of microglia in this disease.

## 2. Methods

### 2.1. Study Design

A case–control study was designed to compare the expression of previously described homeostatic microglia-related genes (*TMEM119*) [19] and genes selected following the literature review [20,21,22,23,24] that play a key role in the acquisition of DAM or MGnD status in neurodegenerative diseases (especially AD), such as *TREM2*, *APOE*, *LPL*, *SPP1*, *TYROBP*, *CD33* and *MS4A* in the postmortem spinal cord of 20 ALS-TDP43 patients and 18 non-neurodegenerative control donors.

### 2.2. Spinal Cord Samples

The Navarrabiomed Brain Bank provided postmortem samples of fresh frozen cervical spinal cord tissue (anterior horn) from 20 patients with sporadic ALS and 18 controls without neurodegenerative disease, following the guidelines of the Spanish legislation [25] on research. 18 out of 20 patients and 16 out of 18 controls are shared with the sample set of a previous microglia-related work developed by our group [26].

The selection of patients was carried out by trained neurologists based on the inclusion criteria as follows: for the ALS group, clinically and neuropathologically diagnosed ALS with TDP-43 deposition were included [27]; for the control group, age- and sex-matched donors without neurodegenerative disease, recent vascular cerebral disease, infection or CNS injury were selected (Table 1).

### 2.3. Neuropathological Examination

Brain processing was performed according to the recommendation guide proposed by BrainNet Europe [28]. Formalin-fixed, paraffin-embedded tissue sections from each region of interest were sectioned at 3–5 μm and counterstained with hematoxylin-eosin for immunohistochemistry analysis with the anti-phospho TDP-43 monoclonal antibody (1:80,000, p5409/410, Cosmo Bio, Otaru, Hokkaido, Japan), mouse monoclonal antibody anti-human PHF-TAU (clone AT-8, Innogenetics, Ghent, Belgium), mouse monoclonal (S6F/3D) anti Beta-amyloid (Leica, Wetzlar, Germany) and mouse monoclonal antibody against α-synuclein (NCL-L-ASYN; Leica Biosystems, Wetzlar, Germany) and were visualized using an automated slide immunostainer (Leica Bond Max, Melbourne, VIC, Australia) with Bond Polymer Refine Detection (Leica Biosystems Newcastle Ltd., Newcastle, UK). Brain and spinal cord sections were stained with Luxol fast blue, rabbit polyclonal antibody anti-Iba1 (1:2000; Wako) and mouse monoclonal prediluted antibody anti-CD68 (1:1; Master Diagnostic) for the study of myelin pathology and inflammatory infiltration.

### 2.4. RNA Isolation from Frozen Spinal Cord Samples

Anterior horn total RNA was isolated from spinal cord homogenates with the RNeasy Lipid Tissue Mini kit (QIAGEN, Venlo, The Netherlands), according to the manufacturer’s recommendations. Recombinant DNase (TURBO DNA-free™ Kit, Ambion, Inc., Austin, TX, USA) was used to remove genomic DNA. The integrity of the RNA was checked in 1.25% agarose gel electrophoresis under denaturing conditions. Both RNA concentration and purity were evaluated using a NanoDrop spectrophotometer. Only those RNA samples with a minimum quality index (260 nm/280 nm absorbance ratios between 1.8 and 2.2, and 260 nm/230 nm absorbance ratios higher than 1.8) were considered in the study.

### 2.5. Reverse Transcription and Gene mRNA Expression Analysis via RT-qPCR

Complementary DNA (cDNA) was reverse transcribed from 1500 ng total RNA with SuperScript^®^ III First-Strand Synthesis Reverse Transcriptase (Invitrogen, Carlsbad, CA, USA) after priming with oligo-d (T) and random primers. Real time quantitative PCR (RT-qPCR) reactions were performed in triplicate with Power SYBR Green PCR Master Mix (Invitrogen, Carlsbad, CA, USA) in a QuantStudio 12K Flex Real-Time PCR System (Applied Biosystems, Foster City, CA, USA) and then repeated within independent cDNA sets to confirm the obtained results [26]. Sequences of primer pairs were designed using Real Time PCR tool (IDT, Coralville, IA, USA) and are listed in Table 2. Relative mRNA expression levels of the studied genes in a particular sample were calculated as previously described [29] and the geometric mean of *ACTB* and *GAPDH* genes was used as reference to normalize expression values.

### 2.6. Statistical Data Analysis

Statistical analysis was performed with SPSS 25.0 (IBM, Inc., Chicago, IL, USA). Prior to differential analysis, continuous variables were tested for normal distribution using the one-sample Kolmogorov–Smirnov test and quantile–quantile (QQ) normal plots. Qualitative variables for each group are shown as a percentage. Quantitative variables are expressed as mean (standard deviation [SD]) when following a normal distribution. The ratio of the mean relative expression in patients to the mean relative expression in controls, or fold change (FC), was established as a measure of the magnitude of change. Differences between cases and controls were explored using Mann–Whitney U test. GraphPad Prism version 6.00 for Windows (GraphPad Software, La Jolla, CA, USA) was used to plot the graphs.

## 3. Results

### 3.1. Sample Composition

A cohort of neuropathological characterized ALS patients (20 cases) and controls (18 cases) was selected from the Navarrabiomed Brain Bank. All ALS cases demonstrated upper and lower motor neuron degeneration accompanied by p-TDP43 neuronal inclusions. To controls selection, patients’ clinical data were evaluated to exclude subjects with a history of cancer. Those who had suffered a stroke or severe head trauma or the presence of brain anomalous proteic aggregates such as β-amyloid or Tau were also excluded.

### 3.2. Comparison of Gene Expression Levels

To determine whether selected genes related to homeostatic microglia (*TMEM119*) and DAM state (*TREM2*, *TYROBP*, *APOE*, *MS4A*, *CD33*, *LPL* and *SPP1*) were differentially expressed in the spinal cord of ALS patients versus controls, mRNA expression levels were measured via RT-qPCR in a cohort of 20 neuropathologically characterized ALS patients and 18 control donors.

**Figure 1:**
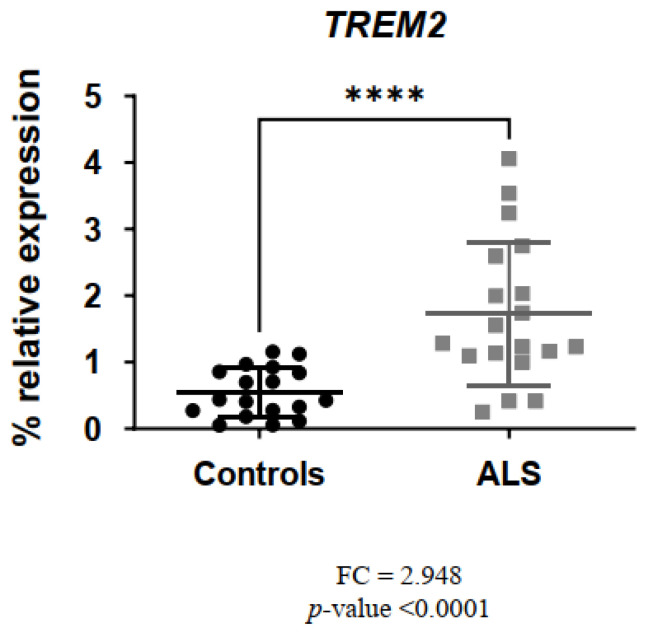
*TREM2* levels were significantly increased in ALS patients versus controls. **** *p*-value < 0.0001.

**Figure 2:**
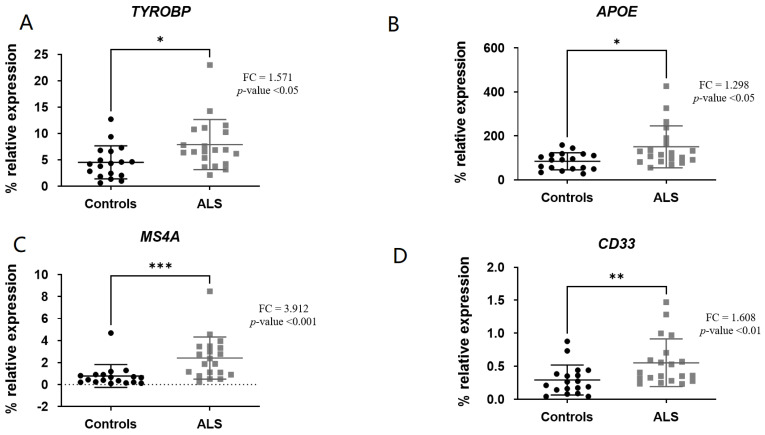
TYROBP (**A**), APOE (**B**), MS4A (**C**) and CD33 (**D**) levels were significantly increased in ALS patients versus controls. * *p*-value < 0.05, ** *p*-value < 0.01, *** *p*-value < 0.001.

The genes involved in facilitating the *TREM2* signaling pathway (*TYROBP*, *APOE*) as well as *MS4A* and *CD33* mRNA levels were significantly increased in ALS patients versus controls: *TREM2* (fold change (FC) = 2.948; *p*-value < 0.0001) (Figure 1), *TYROBP* (FC = 1.571; *p*-value < 0.05) (Figure 2A), *APOE* (FC = 1.298; *p*-value < 0.05) (Figure 2B), *MS4A* (FC = 3.912; *p*-value < 0.001) (Figure 2C) and *CD33* (FC = 1.608; *p*-value < 0.01) (Figure 2D).

**Figure 3:**
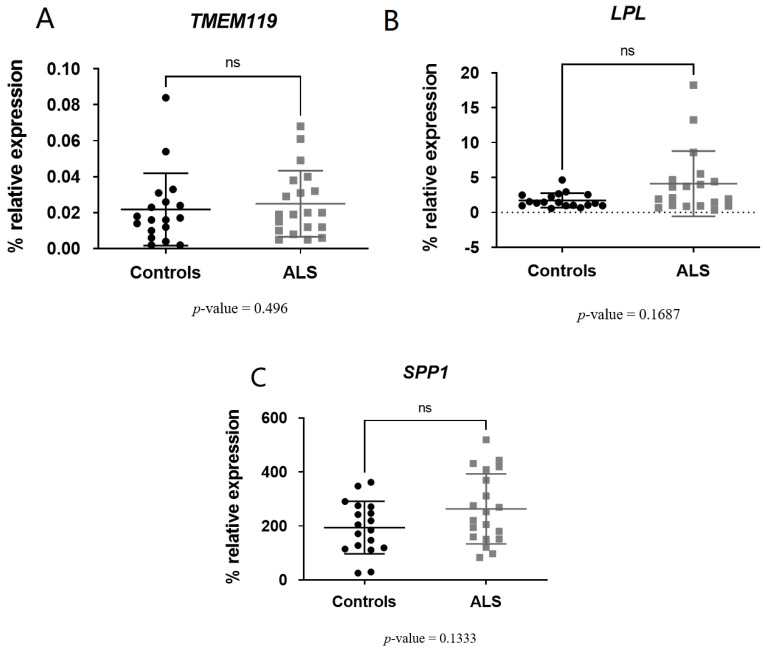
TMEM119 (**A**), LPL (**B**) and SPP1 (**C**) levels were not significant in ALS patients versus controls. ns or non-significant means *p*-value is >0.05.

However, we found no significant differences in the expression of genes related to homeostatic microglia: *TMEM119* (*p*-value = 0.496) (Figure 3A) and genes related to the late state of DAM [8]: *LPL* (*p*-value = 0.1687) (Figure 3B) and *SPP1* (*p*-value = 0.1333) (Figure 3C).

## 4. Discussion

Studies based on gene expression signatures across several diseases in human brain and animal models, have observed diverse states of microglia that play different roles throughout the different stages of neurodegenerative diseases [16]. The key feature of DAM, present at sites of neurodegeneration, is the downregulation of genes related to homeostatic microglia and the upregulation of genes involved in phagocytic, lysosomal and lipid metabolism pathways [9,30]. Moreover, the transition between homeostatic microglia and DAM is known to be mediated by the expression of *TREM2* [10].

Currently, the understanding of the role of DAM in ALS is limited. In the present study, we have attempted to approach the microglial state in ALS by analyzing the expression of key genes related to the homeostatic state (*TMEM119*) or required for induction to the neurodegenerative DAM state according to previous descriptions (*TREM2*, *APOE*, *TYROBP*, *CD33*, *MS4A*, *LPL*, *SPP1*) [8].

We have showed that *TMEM119* was not differentially expressed in the spinal cord of ALS patients versus controls. *TMEM119* is a gene that encodes for a transmembrane protein 119 and has been proposed as a microglial marker based on its specific expression in homeostatic microglia, but not in other brain-resident cells nor in infiltrating macrophages [31]; however, its use as a consistent marker of microglia is still controversial [32]. The lack of upregulation of *TMEM119* observed in our study agrees with a recent work [33] that suggests *TMEM119−* microglia is associated with neurodegenerative DAM in ALS, while *TMEM119+* microglia may be associated with DAM-independent neurodegeneration.

Regarding the specific genes enriched in microglia that we have analyzed in this study, and that according to previous data are highly expressed in the DAM state, we observed upregulation of all of them except *LPL* and *SPP1*. Microglia are dependent on *TREM2* expression to completely adopt a DAM profile [10], and interestingly, this study detected that *TREM2* mRNA was upregulated in spinal cord ALS patients versus controls, which reinforces our previous observations [26] conducted on a different sample set that shared several cases and controls used in this study. *TREM2* belongs to a family of transmembrane receptors of innate immune system and it is expressed in the membrane of myeloid cells, dendritic cells, osteoclasts and microglia [17]. *TREM2* plays a critical role in microglial function, including phagocytosis, cytokine production and cell survival, and genetic evidence has linked *TREM2* to neurodegenerative diseases including ALS, but its function in ALS pathogenesis is largely unknown [34,35,36,37,38]. Most likely, the function of microglial *TREM2* may vary during the disease progression [36].

Genome-wide association studies discovered, in addition to *TREM2*, the overexpressed genes in the DAM state selected for this study, *TYROBP* [39], *APOE* [40,41,42,43], *CD33* [44,45,46,47] and *MS4A* [23,45], as AD-associated microglial risk genes [48], but there is less evidence for its involvement in ALS [17,49]. Our study showed that mRNA levels of *TYROBP*, *APOE*, *MS4A* and *CD33* were significantly increased in the spinal cord of ALS patients versus controls. APOE and TYROBP (DAP12) have been reported to act as a ligand of TREM2 by enhancing its signaling and promoting the conversion of homeostatic microglia to DAM in mouse models of ALS [17]. CD33 and MS4A are transmembrane immunoreceptors expressed on microglia. *CD33* has been identified as an AD susceptibility factor [44,50] and it has been positioned as a potential therapeutic target [51]. The *MS4A* gene cluster is a key modulator of soluble TREM2 that has been implicated in AD and progressive supranuclear palsy (PSP) [52] pathogenesis and might provide new treatment opportunities [23,53]. To our knowledge, the contribution of *CD33* and *MS4A* in the pathogenesis of ALS has not been previously documented.

The DAM signature described in AD disease also overexpresses *LPL* and *SPP1* [14], genes related to late state of DAM [8]. However, our results do not show a significant overexpression of *LPL* or *SPP1* in the late state of the disease, which could suggest that there may be an incomplete or truncated expression of the DAM program in ALS.

The *SPP1* gene encodes osteopontin (OPN), a protein that has aroused interest as a therapeutic target in several immune and inflammatory diseases, including multiple sclerosis as a neurological condition [54]. OPN is a glycoprotein that is expressed in different cell types, among them microglia, which represents one of the main sources of OPN in the CNS. OPN facilitates microglia-mediated phagocytosis and repair function through different mechanisms. The role of OPN is controversial, neuroprotective or neurotoxic depending on the context and the activation state of microglia, and it seems that dysfunctional OPN could be involved in several neurodegenerative diseases including ALS [55].

Regarding *LPL,* it encodes lipoprotein lipase that regulates microglial lipid metabolism. It has been shown that microglia lacking *LPL* have altered lipid metabolism and polarize towards a pro-inflammatory state [55]. In addition, lipid accumulation of myelin debris in microglia (LDAM) impairs their phagocytic function and contributes to the progression of neurodegenerative disease [56].

We could speculate whether dysfunctional osteopontin and lipid metabolism dysfunction due to reduced expression of SPP1 and LPL, respectively, could mediate reduced the phagocytic capacity of microglia-DAM in ALS.

It is possible that the DAM state is not enough to explain the functional complexity of the role of microglia in ALS, but we found it especially noteworthy that the genes related to the early-state DAM description [7,8,9] were overexpressed, and yet, we did not find differences in the genes related to late-state DAM (*SPP1* and *LPL*), which could suggest a partial expression of the DAM program in ALS.

Our study is limited by the small number of genes analyzed. Moreover, we have studied postmortem spinal cord tissue from patients with end-stage ALS, and it is likely that the status of microglia varies throughout the course of ALS acquiring different transcriptional profiles that understandably could not be characterized in our study. As a result, the potential use of these findings as early biomarkers of ALS is thus limited. Our results define a gene expression profile with upregulation of DAM-related genes such as *TREM2*, *TYROBP*, *APOE*, *CD33* and *MS4A* and not of microglia-related homeostatic genes (TMEM119), thus supporting that DAM status plays a key role in the neuroinflammatory changes occurring in the spinal cord of ALS patients. It is necessary future studies to investigate the dynamic evolution of the microglial state in the disease, in order to identify therapeutic targets that can modify the inflammatory response at earlier stages of ALS.

In conclusion, the upregulation of *TREM2*, *APOE*, *CD33*, *TYROBP* and *MS4A* in the spinal cord of ALS could be potential markers of the presence of the DAM state in ALS in addition to these genes positioning themselves as potential therapeutic targets in the disease. We also showed that *C33* and *MS4A* may play a role in the physiopathology of ALS, not previously described. Despite the limitations of this preliminary study, we hope that our results will stimulate new studies deepening the role of the DAM state in ALS.

## Figures and Tables

**Table 1:** Data from all participants.

	ALS	Controls	*p*-Value
Spinal cord donors			
*n*	20	18	
Age (years) (SD) ^1^	70.1 (±13.79)	73.3 (±14.29)	0.491
Sex (F/M)	12/8	6/12	0.093
PMI ^2^ (h); median (range)	6.7 (3–12)	6.9 (2–16)	0.734

^1^ Standard deviation; ^2^ Postmortem interval.

**Table 2:** RT-qPCR primers used in the study.

ID	Accesion Number ^1^	Amplicon Size (bp ^2^)	Tm	Forward Primer	Tm2 ^3^	Reverse Primer
*TREM2*	NM_018965	116	61.0	CTGCTCATCTTACTCTTTGTCAC	62.3	CAGTGCTTCATGGAGTCATAGG
*TYROBP*	NM_003332	146	61.6	CTGGCCGTGTACTTCCTG	62	GTGTTGAGGTCGCTGTAGAC
*APOE*	NM_000041	143	62.2	TTGCTGGTCACATTCCTGG	62.2	AGGTAATCCCAAAAGCGACC
*MS4A*	NM_148975	148	61.9	TCTTGAAGGGAGAACCCAAAG	62	CCCCAAATTGTGTACCCGATA
*CD33*	NM_001772	132	61.9	TGTCAGGTGAAGTTCGCTG	61.8	TGCTCTGGTCTCTTGTTTCC
*TMEM119*	NM_181724	111	61.7	CCACTCTCGCTCCATTCG	61.6	CAGCAACAGAAGGATGAGGA
*LPL*	NM_000237	129	61.9	AAAGTGTCTCATTTGCAGAAAGG	61.9	CACAGAATTCACATGCCGTTC
*SPP1*	NM_001040058	148	61.8	GTCCCCACAGTAGACACATATG	62.1	TCAACTCCTCGCTTTCCATG

^1^ Amplified transcripts are identified according to RefSeq Accession number; ^2^ bp: base pair; ^3^ Tm: melting temperature.

## Data Availability

The data presented in this study are available on request from the corresponding author.
